# Design of microarray probes for virus identification and detection of emerging viruses at the genus level

**DOI:** 10.1186/1471-2105-7-232

**Published:** 2006-04-28

**Authors:** Cheng-Chung Chou, Te-Tsui Lee, Chun-Houh Chen, Hsiang-Yun Hsiao, Yi-Ling Lin, Mei-Shang Ho, Pan-Chyr Yang, Konan Peck

**Affiliations:** 1 Center for Genomic Medicine, National Taiwan University, Taipei, 100, ROC; 2 Institute of Biomedical Sciences, Academia Sinica, Taipei, Taiwan, 115, ROC; 3 Institute of Statistical Science, Academia Sinica, Taipei, Taiwan, 115, ROC

## Abstract

**Background:**

Most virus detection methods are geared towards the detection of specific single viruses or just a few known targets, and lack the capability to uncover the novel viruses that cause emerging viral infections. To address this issue, we developed a computational method that identifies the conserved viral sequences at the genus level for all viral genomes available in GenBank, and established a virus probe library. The virus probes are used not only to identify known viruses but also for discerning the genera of emerging or uncharacterized ones.

**Results:**

Using the microarray approach, the identity of the virus in a test sample is determined by the signals of both genus and species-specific probes. The genera of emerging and uncharacterized viruses are determined based on hybridization of the viral sequences to the conserved probes for the existing viral genera. A detection and classification procedure to determine the identity of a virus directly from detection signals results in the rapid identification of the virus.

**Conclusion:**

We have demonstrated the validity and feasibility of the above strategy with a small number of viral samples. The probe design algorithm can be applied to any publicly available viral sequence database. The strategy of using separate genus and species probe sets enables the use of a straightforward virus identity calculation directly based on the hybridization signals. Our virus identification strategy has great potential in the diagnosis of viral infections. The virus genus and specific probe database and the associated summary tables are available at

## Background

New human viral pathogens, such as the etiologic agent causing severe acute respiratory syndrome (SARS) and avian influenza, continue to emerge and have become major health problems worldwide. Traditional viral detection techniques such as *in vitro *viral cultures, immunologic assays, and the PCR method [1] can identify only one or just a few specific viral targets in a single test. When presented with patients having symptoms of unknown etiology, multiple diagnostic assays are often performed in parallel, which is a time-consuming and labor-intensive process.

Oligonucleotide microarrays have been used for monitoring and analyzing viral pathogens [2-5]. There are currently two types of DNA microarrays used for virus identification: (i) those based on using short oligonucleotide probes that are sensitive to single-base mismatches, which are used to detect or identify subtypes of known viruses; and (ii) those based on using long oligonucleotide probes to uncover the identity of the virus present in a sample. For the former application, the entire set of probes on the microarray is designed to detect the mutations in the viral genome to allow one specific viral species and its subtypes to be discerned. An example of this application is the virus microarrays for discriminating different subtypes of influenza viruses [4,5]. For the latter application, long oligonucleotide probes (60- or 70-mers) that tolerate sequence mismatches are used for broad detection specificity to a large number of viruses. This study focuses on the latter application: uncovering the identity of viruses present in a sample rather than detecting the mutations of a specific known virus.

Previously reported studies [2,3] have employed the tiling approach (70-nt segments with 25-nt offsets) to design microarray probes for uncovering the identities of viruses in a sample, using the BLASTN sequence alignment program to select the most similar sequences in the respective viral family of each sequenced viral genome. The unknown or unsequenced members of existing viral families can be detected by hybridization to the microarray elements with sequence similarity. Conserved sequences were selected based only on the unidirectional relationship between one genome and the others in the same viral family. A substantial percentage of sequence redundancy exists among these family probes, and a family probe set usually contains a large number of redundant conserved probes. For virus identification, microarray data have been categorized into individual viral families and converted to a linear visualization scale (a bar-coded linear pattern) [2] or an alignment of the probe sequences giving hybridization signal intensities has been employed to determine the true identity of specific viruses [3].

Based on the experiences of identifying novel viruses, such as the SARS coronavirus (SARS-CoV) or the Nipah virus, a virus can exhibit considerable sequence identity with other viruses in the same genus [6,7]. Although long probes possess a high hybridization efficiency, they often exhibit poor discrimination of specific viruses. Therefore, significant cross-hybridization of a viral genome to the highly similar noncognate long probes is expected. Thus, identifying viral species using the previously reported viral microarray probe design strategy requires additional intensive computation to analyze the complex hybridization patterns. A software program for implementing such a virus identification strategy has been developed [8]. The program determines the identity of specific virus(es) in a test sample by comparing observed patterns of microarray hybridization intensities with a set of theoretical hybridization energy profiles as computed from the alignments of microarray oligonucleotides against all completely sequenced reference viral genomes available in the GenBank database.

In order to design a virus microarray capable of identifying a larger number of viruses with high accuracy but with fewer probes than previously reported, we have developed a new method for designing probes for the precise and direct identification of viruses at the genus, species, and strain levels. Our method narrows down the computation of conserved sequences to the genus level and eliminates sequence redundancy within a viral genus probe set by considering the mutual sequence similarity among all the viral genomes in the same genus. Compared with the tiling approach, our approach greatly reduces the redundancy and number of conserved probes for a given viral genus. The method involves two separate sets of probes: (i) the conserved sequence probes for genus identification; and (ii) specific probes for viral species or stain identification. The probe set for each genus contains a panel of 70-mer oligonucleotides that not only covers all the sequenced known viruses of the genus but also maximizes the likelihood of detecting emerging or unsequenced viruses. Long 70-mer probes help to alleviate sequence-dependent hybridization variation, improve hybridization efficiency, and enhance the capability of identifying unknown viruses. To compensate for the poor discrimination capability of long probes, additional species- or subtype-specific probes were designed for each fully sequenced virus to enhance the specificity of virus identification. The identity of a virus is determined based on a simple arithmetic algorithm that calculates the average signal intensity of the multiple probes for a virus, in contrast to the intensive computation needed for pattern comparisons. The use of separate genus and species probe sets facilitates a cross-examination strategy to increase the identification accuracy. The identity of a virus is established by the concordant results for both the genus- and cognate-species-specific probes.

## Results

### Viral sequences for computation

More than 19,600 full-length viral genomes covering 53 viral families and 214 genera were obtained from ~ 340,000 viral sequences archived in the GenBank viral database (Release 152). The genome segments of the viruses with segmented or multipartite genomes were treated as individual viral sequences. Additional partially sequenced viral genomes were included for computing the conserved genus probes for genera with ≤ 5 fully sequenced members and viruses with segmented or multipartite genomes, which made up a total number of 27,610 viral genome sequences for the computation. Viral genome sequences with more than 99.5% similarity were treated as redundant, and only the longest sequences were retained; the resulting 5,749 nonredundant viral genome sequences were used in the analysis.

### Algorithm for designing the conserved probe

The probe design strategy employs two separate probe sets: species-specific probes and conserved genus probes. Genera with insufficient viral sequences for identifying conserved probe sequences are marked in the virus probe database and extra species-specific probes are designed for each viral species of each such genus. The species-specific probes were designed using the algorithm described by Chang and Peck [9], and the conserved genus probes for each viral genus were designed according to the following guidelines:

1. A viral genus *G *= {*v*_*i*_| *i *= 1,..., *n*} is a collection of *n *viruses, in which each virus *v*_*i *_(*i *= 1,...,* n*) is associated with a subset of *G*, where subset *G*^(*i*) ^≡ *G*-{*v*_*i*_}.

2. Given a virus *v*_*i *∈_*G*, the BLASTN result for the comparison of this virus with another virus in the same genus, *v*_*j *∈_*G*^(*i*)^, can be expressed as an indicator function *I*_*ji*_:

Iji(x)={1,x∈similarity sequence segments of vj and vi0,otherwise,
 MathType@MTEF@5@5@+=feaafiart1ev1aaatCvAUfKttLearuWrP9MDH5MBPbIqV92AaeXatLxBI9gBaebbnrfifHhDYfgasaacH8akY=wiFfYdH8Gipec8Eeeu0xXdbba9frFj0=OqFfea0dXdd9vqai=hGuQ8kuc9pgc9s8qqaq=dirpe0xb9q8qiLsFr0=vr0=vr0dc8meaabaqaciaacaGaaeqabaqabeGadaaakeaacqWGjbqsdaWgaaWcbaGaemOAaOMaemyAaKgabeaakmaabmaabaGaemiEaGhacaGLOaGaayzkaaGaeyypa0ZaaiqaaeaafaqaaeGabaaabaGaeGymaeJaeiilaWIaemiEaGNaeyicI4Saee4CamNaeeyAaKMaeeyBa0MaeeyAaKMaeeiBaWMaeeyyaeMaeeOCaiNaeeyAaKMaeeiDaqNaeeyEaKNaeeiiaaIaee4CamNaeeyzauMaeeyCaeNaeeyDauNaeeyzauMaeeOBa4Maee4yamMaeeyzauMaeeiiaaIaee4CamNaeeyzauMaee4zaCMaeeyBa0MaeeyzauMaeeOBa4MaeeiDaqNaee4CamNaeeiiaaIaee4Ba8MaeeOzayMaeeiiaaIaemODay3aaSbaaSqaaiabdQgaQbqabaGccqqGGaaicqqGHbqycqqGUbGBcqqGKbazcqqGGaaicqWG2bGDdaWgaaWcbaGaemyAaKgabeaaaOqaaiabicdaWiabcYcaSiabb+gaVjabbsha0jabbIgaOjabbwgaLjabbkhaYjabbEha3jabbMgaPjabbohaZjabbwgaLbaaaiaawUhaaiabcYcaSaaa@7EFE@

where *x *is any sequence segment within the genome of *v*_*i*_, and the similarity sequence segment is any segment of virus *v*_*i *_having either (i) more than 75% local sequence similarity in a 50-bp window with any virus *v*_*j *∈_*G*^(*i*)^, as determined using the identity percentage calculation in the BLASTN program (equivalent to BLASTN sequence identity matches of 38-bp); or (ii) >15 consecutive bases pairing with *v*_*j *_[10].

3. The sequence segments of virus *v*_*i *_similar to other viruses in *G*^(*i*) ^are then defined as the summation of all these indicator functions,*S*_*i*_(*x*) = ∑_*j≠i*_* I*_*ji*_(*x*), which is a step contour function from 0 to *n*-1.

4. The conserved sequences of *v*_*i *_are defined as the regions with the maximum number of similarity hits in *S*_*i*_(*x*): *C*_*i *_= {*x*| *S*_*i*_(*x*) = max⁡x
 MathType@MTEF@5@5@+=feaafiart1ev1aaatCvAUfKttLearuWrP9MDH5MBPbIqV92AaeXatLxBI9gBaebbnrfifHhDYfgasaacH8akY=wiFfYdH8Gipec8Eeeu0xXdbba9frFj0=OqFfea0dXdd9vqai=hGuQ8kuc9pgc9s8qqaq=dirpe0xb9q8qiLsFr0=vr0=vr0dc8meaabaqaciaacaGaaeqabaqabeGadaaakeaadaWfqaqaaiGbc2gaTjabcggaHjabcIha4bWcbaGaemiEaGhabeaaaaa@3284@ (*S*_*i*_(*x*))}.

5. The conserved sequence set of *G *is the union of all *C*_*i*_'*s*: *C'*(*G*) = ∪i=1nCi
 MathType@MTEF@5@5@+=feaafiart1ev1aaatCvAUfKttLearuWrP9MDH5MBPbIqV92AaeXatLxBI9gBaebbnrfifHhDYfgasaacH8akY=wiFfYdH8Gipec8Eeeu0xXdbba9frFj0=OqFfea0dXdd9vqai=hGuQ8kuc9pgc9s8qqaq=dirpe0xb9q8qiLsFr0=vr0=vr0dc8meaabaqaciaacaGaaeqabaqabeGadaaakeaadaWeWbqaaiabdoeadnaaBaaaleaacqWGPbqAaeqaaaqaaiabdMgaPjabg2da9iabigdaXaqaaiabd6gaUbqdcqWIQisvaaaa@35C0@. To eliminate sequence redundancy, the longest sequence segment *C*_*L *_(the query sequence) in *C'*(*G*) is selected first and aligned against the other sequences in it using the BLASTN program. Any sequences in *C'*(*G*)-{*C*_*L*_} having ≥ 80% similarity with *C*_*L *_are grouped together with *C*_*L *_to form *C'*_1_(*G*), the first subgroup of *C'*(*G*). *C*_*L *_is then renamed as *C*_(1) _to represent the first subgroup. The first subgroup,*C'*_1_(*G*), is then excluded from *C'*(*G*). This procedure is repeated for the remaining sequence segments in *C'*(*G*) until *k *such longest sequence segments {*C*_(1)_*,..., C*_(*k*) _and *k *subgroups {*C'*_1_(*G*)*,..., C'*_*k*_(*G*)} are identified, with every sequence in *C'*(*G*) assigned to one subgroup. The union of all the longest sequences, ∪i=1kC(i)
 MathType@MTEF@5@5@+=feaafiart1ev1aaatCvAUfKttLearuWrP9MDH5MBPbIqV92AaeXatLxBI9gBaebbnrfifHhDYfgasaacH8akY=wiFfYdH8Gipec8Eeeu0xXdbba9frFj0=OqFfea0dXdd9vqai=hGuQ8kuc9pgc9s8qqaq=dirpe0xb9q8qiLsFr0=vr0=vr0dc8meaabaqaciaacaGaaeqabaqabeGadaaakeaadaWeWbqaaiabdoeadnaaBaaaleaadaqadaqaaiabdMgaPbGaayjkaiaawMcaaaqabaaabaGaemyAaKMaeyypa0JaeGymaedabaGaem4AaSganiablQIivbaaaa@3743@, represents the nonredundant conserved sequence set of *G*. A simplified illustration delineating the above procedures is provided in Additional File 1.

6. To prevent significant cross-hybridization to noncognate viral genus sequences, a 70-mer conserved probe set *P *= {*p*_*j*_| *j *= 1,..., *m *is selected from each nonredundant sequence in ∪i=1kC(i)
 MathType@MTEF@5@5@+=feaafiart1ev1aaatCvAUfKttLearuWrP9MDH5MBPbIqV92AaeXatLxBI9gBaebbnrfifHhDYfgasaacH8akY=wiFfYdH8Gipec8Eeeu0xXdbba9frFj0=OqFfea0dXdd9vqai=hGuQ8kuc9pgc9s8qqaq=dirpe0xb9q8qiLsFr0=vr0=vr0dc8meaabaqaciaacaGaaeqabaqabeGadaaakeaadaWeWbqaaiabdoeadnaaBaaaleaadaqadaqaaiabdMgaPbGaayjkaiaawMcaaaqabaaabaGaemyAaKMaeyypa0JaeGymaedabaGaem4AaSganiablQIivbaaaa@3743@ based on the following criteria: (i) GC content between 40% and 60%, (ii) <5 continuous mononucleotide repeats, (iii) <25-bp BLASTN sequence identity matches, and (iv) ≤ 15 consecutive bases pairing with other viral sequences in the noncognate viral genus. The probes exhibiting sequence overlap are further screened using the Mfold program [11] to pick the one that has the maximal Gibbs free energy of the secondary structure, such that there is no sequence overlap among *P*.

7. To search for a subset of *P *with the minimum number of probes that can collectively yield similarity hits with all the virus members of *G*, each *p*_*j *∈_*P *is aligned against *v*_*i *∈_*G *using the BLASTN program. The BLASTN result between *p*_*j *_and *v*_*i *_can be expressed as an array,*A*_*j × i*_:

Aj×i={hit, aji∈pj having>38−bp BLASTN identify matches to vino hit, otherwise.
 MathType@MTEF@5@5@+=feaafiart1ev1aaatCvAUfKttLearuWrP9MDH5MBPbIqV92AaeXatLxBI9gBaebbnrfifHhDYfgasaacH8akY=wiFfYdH8Gipec8Eeeu0xXdbba9frFj0=OqFfea0dXdd9vqai=hGuQ8kuc9pgc9s8qqaq=dirpe0xb9q8qiLsFr0=vr0=vr0dc8meaabaqaciaacaGaaeqabaqabeGadaaakeaacqWGbbqqdaWgaaWcbaGaemOAaOMaey41aqRaemyAaKgabeaakiabg2da9maaceaabaqbaeaabiqaaaqaaiabbIgaOjabbMgaPjabbsha0jabbYcaSiabbccaGiabdggaHnaaBaaaleaacqWGQbGAcqWGPbqAaeqaaOGaeyicI4SaemiCaa3aaSbaaSqaaiabdQgaQbqabaGccqqGGaaicqqGObaAcqqGHbqycqqG2bGDcqqGPbqAcqqGUbGBcqqGNbWzcqGH+aGpcqaIZaWmcqaI4aaocqGHsislcqqGIbGycqqGWbaCcqqGGaaicqqGcbGqcqqGmbatcqqGbbqqcqqGtbWucqqGubavcqqGobGtcqqGGaaicqqGPbqAcqqGKbazcqqGLbqzcqqGUbGBcqqG0baDcqqGPbqAcqqGMbGzcqqG5bqEcqqGGaaicqqGTbqBcqqGHbqycqqG0baDcqqGJbWycqqGObaAcqqGLbqzcqqGZbWCcqqGGaaicqqG0baDcqqGVbWBcqqGGaaicqWG2bGDdaWgaaWcbaGaemyAaKgabeaaaOqaaiabb6gaUjabb+gaVjabbccaGiabbIgaOjabbMgaPjabbsha0jabbYcaSiabbccaGiabb+gaVjabbsha0jabbIgaOjabbwgaLjabbkhaYjabbEha3jabbMgaPjabbohaZjabbwgaLbaaaiaawUhaaiabc6caUaaa@8E6F@

Thus, the 70-mer conserved probe set can be redefined as *P *= {*p*_*j*_| *j *= 1,..., *m*}, *p*_*j *⊆_*G'*, and *G' *= {*v'*_*i*_| *i *= 1,..., *m*, where *v' *_*i *_represents similarity hit(s) with *v*_*i *_by *p*_*j*(*s*) ∈_*P*. Let *Card*(*p*_*j*_) be the cardinality of probe *p*_*j *_(i.e., the number of similarity hits with probe *p*_*j*_) in array *A*_*j × i*_. We first search for a minimum coverage set Ps*
 MathType@MTEF@5@5@+=feaafiart1ev1aaatCvAUfKttLearuWrP9MDH5MBPbIqV92AaeXatLxBI9gBaebbnrfifHhDYfgasaacH8akY=wiFfYdH8Gipec8Eeeu0xXdbba9frFj0=OqFfea0dXdd9vqai=hGuQ8kuc9pgc9s8qqaq=dirpe0xb9q8qiLsFr0=vr0=vr0dc8meaabaqaciaacaGaaeqabaqabeGadaaakeaacqWGqbaudaqhaaWcbaGaem4CamhabaGaeiOkaOcaaaaa@304D@ ⊆ *P *such that *Card*(Ps*
 MathType@MTEF@5@5@+=feaafiart1ev1aaatCvAUfKttLearuWrP9MDH5MBPbIqV92AaeXatLxBI9gBaebbnrfifHhDYfgasaacH8akY=wiFfYdH8Gipec8Eeeu0xXdbba9frFj0=OqFfea0dXdd9vqai=hGuQ8kuc9pgc9s8qqaq=dirpe0xb9q8qiLsFr0=vr0=vr0dc8meaabaqaciaacaGaaeqabaqabeGadaaakeaacqWGqbaudaqhaaWcbaGaem4CamhabaGaeiOkaOcaaaaa@304D@) = min *Card*(*P*_*S *_) for all subsets of *P *(*P*_*S *_⊆ *P*) satisfying the following sequential criteria: (i) (∪∀pj∈Ps*pj)=G′
 MathType@MTEF@5@5@+=feaafiart1ev1aaatCvAUfKttLearuWrP9MDH5MBPbIqV92AaeXatLxBI9gBaebbnrfifHhDYfgasaacH8akY=wiFfYdH8Gipec8Eeeu0xXdbba9frFj0=OqFfea0dXdd9vqai=hGuQ8kuc9pgc9s8qqaq=dirpe0xb9q8qiLsFr0=vr0=vr0dc8meaabaqaciaacaGaaeqabaqabeGadaaakeaadaqadaqaamaatafabaGaemiCaa3aaSbaaSqaaiabdQgaQbqabaaabaGaeyiaIiIaemiCaa3aaSbaaWqaaiabdQgaQbqabaWccqGHiiIZcqWGqbaudaqhaaadbaGaem4CamhabaGaeiOkaOcaaaWcbeqdcqWIQisvaaGccaGLOaGaayzkaaGaeyypa0Jafm4raCKbauaaaaa@3E02@[12,13], (ii) max  if more than one set of Ps*
 MathType@MTEF@5@5@+=feaafiart1ev1aaatCvAUfKttLearuWrP9MDH5MBPbIqV92AaeXatLxBI9gBaebbnrfifHhDYfgasaacH8akY=wiFfYdH8Gipec8Eeeu0xXdbba9frFj0=OqFfea0dXdd9vqai=hGuQ8kuc9pgc9s8qqaq=dirpe0xb9q8qiLsFr0=vr0=vr0dc8meaabaqaciaacaGaaeqabaqabeGadaaakeaacqWGqbaudaqhaaWcbaGaem4CamhabaGaeiOkaOcaaaaa@304D@ having *Card*(Ps*
 MathType@MTEF@5@5@+=feaafiart1ev1aaatCvAUfKttLearuWrP9MDH5MBPbIqV92AaeXatLxBI9gBaebbnrfifHhDYfgasaacH8akY=wiFfYdH8Gipec8Eeeu0xXdbba9frFj0=OqFfea0dXdd9vqai=hGuQ8kuc9pgc9s8qqaq=dirpe0xb9q8qiLsFr0=vr0=vr0dc8meaabaqaciaacaGaaeqabaqabeGadaaakeaacqWGqbaudaqhaaWcbaGaem4CamhabaGaeiOkaOcaaaaa@304D@) = min *Card*(*P*_*S*_), and (iii) randomly pick one of those Ps*
 MathType@MTEF@5@5@+=feaafiart1ev1aaatCvAUfKttLearuWrP9MDH5MBPbIqV92AaeXatLxBI9gBaebbnrfifHhDYfgasaacH8akY=wiFfYdH8Gipec8Eeeu0xXdbba9frFj0=OqFfea0dXdd9vqai=hGuQ8kuc9pgc9s8qqaq=dirpe0xb9q8qiLsFr0=vr0=vr0dc8meaabaqaciaacaGaaeqabaqabeGadaaakeaacqWGqbaudaqhaaWcbaGaem4CamhabaGaeiOkaOcaaaaa@304D@ with the same max  if more than one set of Ps*
 MathType@MTEF@5@5@+=feaafiart1ev1aaatCvAUfKttLearuWrP9MDH5MBPbIqV92AaeXatLxBI9gBaebbnrfifHhDYfgasaacH8akY=wiFfYdH8Gipec8Eeeu0xXdbba9frFj0=OqFfea0dXdd9vqai=hGuQ8kuc9pgc9s8qqaq=dirpe0xb9q8qiLsFr0=vr0=vr0dc8meaabaqaciaacaGaaeqabaqabeGadaaakeaacqWGqbaudaqhaaWcbaGaem4CamhabaGaeiOkaOcaaaaa@304D@ satisfy condition (ii). We then keep searching for other subsets of *P-*Ps*
 MathType@MTEF@5@5@+=feaafiart1ev1aaatCvAUfKttLearuWrP9MDH5MBPbIqV92AaeXatLxBI9gBaebbnrfifHhDYfgasaacH8akY=wiFfYdH8Gipec8Eeeu0xXdbba9frFj0=OqFfea0dXdd9vqai=hGuQ8kuc9pgc9s8qqaq=dirpe0xb9q8qiLsFr0=vr0=vr0dc8meaabaqaciaacaGaaeqabaqabeGadaaakeaacqWGqbaudaqhaaWcbaGaem4CamhabaGaeiOkaOcaaaaa@304D@ with the same criteria until the probe set finally has a twofold coverage of *G*.

### Illustration of the conserved sequence design method

**Figure 1:**
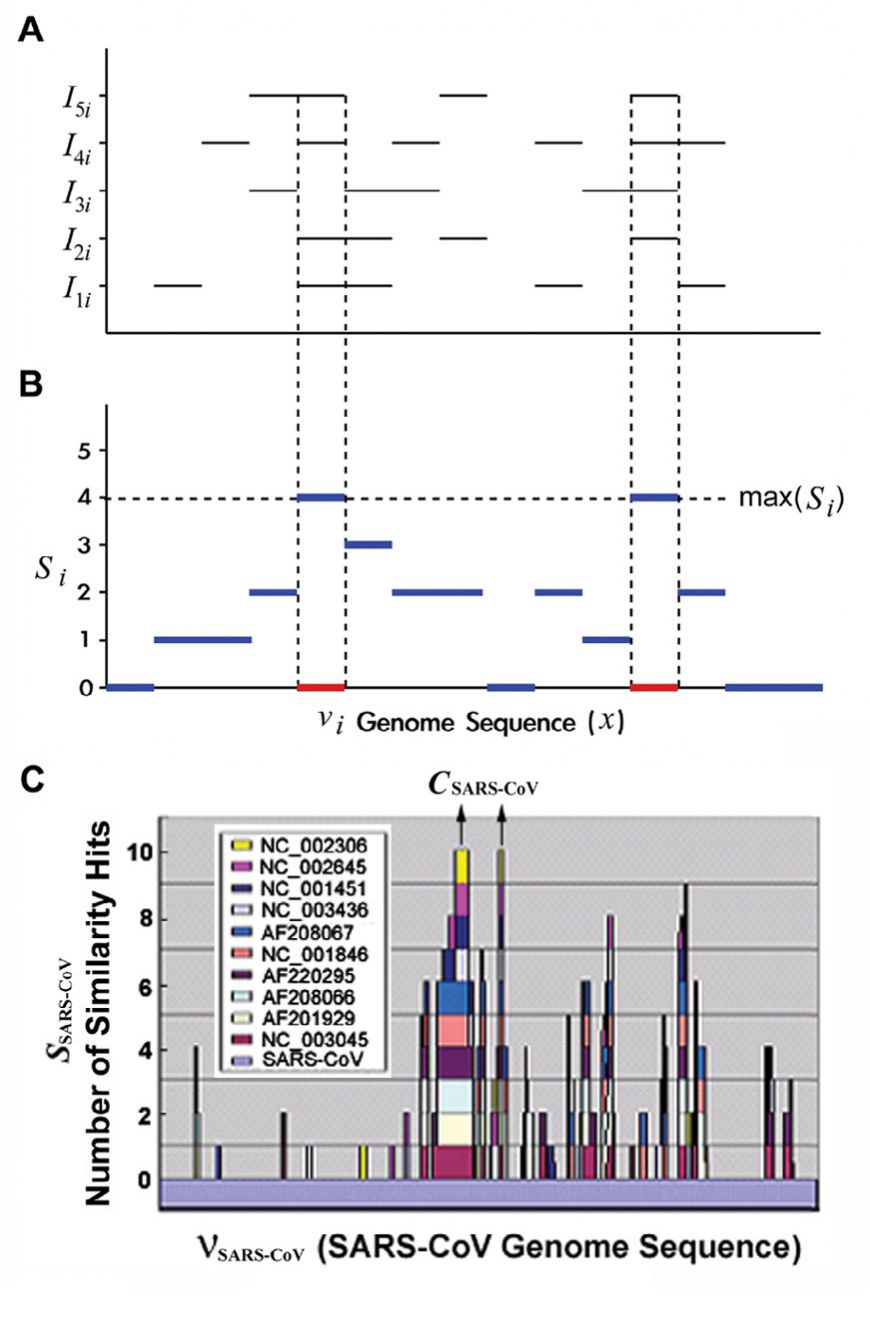
**Algorithm for designing conserved probes**. Schematic illustration of the conserved sequence design method. (A) The similarity sequence segments (*I*_1*i *_to *I*_5*i*_) of a given virus *v*_*i *_are aligned with the sequence segments of the other five viruses within the same viral genus by the BLASTN program to find the regions with high similarity hits. (B) Graph of the numbers of similarity hits, *S*_*i*_, obtained by the procedure illustrated in panel A. The largest number of hits as illustrated is max(*S*_*i*_), which equals 4. The two regions with the highest similarity hits are marked by vertical dotted lines. (C) A case study of locating the conserved sequence segments in coronavirus genus. The genus contains the following 11 fully sequenced viral genomes: NC_002645, human coronavirus 229E; NC_001451, avian infectious bronchitis virus; NC_003436, porcine epidemic diarrhea virus; AF201929, murine hepatitis virus strain 2; AF208067, murine hepatitis virus strain ML-10; AF208066, murine hepatitis virus strain Penn 97-1; NC_001846, murine hepatitis virus strain A59; NC_003045, bovine coronavirus; AF220295, bovine coronavirus strain Quebec; NC_002306, transmissible gastroenteritis virus; and SARS-CoV, SARS coronavirus.

To demonstrate the conserved sequence design method, a simplified example of the procedure is shown in Figure 1A. Assuming that there are six (*n*) viruses in a viral genus (*G*), the similarity sequence segments for a given virus *v*_*i *_compared to the other five (*n*-1) viral genomes (*G*^(*i*)^) are designated *I*_*ji *_(*j *= 1,..., 5) (Fig. 1A). These similarity segments are then ranked by the number of similarity hits with other viral genomes (*S*_*i*_) and plotted along the *v*_*i *_genome. The highest-ranking (max(*S*_*i*_)) similarity sequences should be the most-conserved sequences (*C*_*i*_) for virus *v*_*i *_and the other five viral genomes (red segments in Fig. 1B).

**Figure 2:**
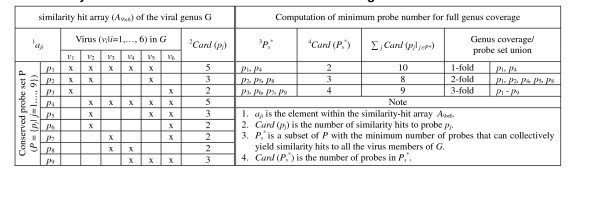
Table 1 - Example searching the minimum number of probes that generate similarity hits with all the virus members within a viral genus.

The above procedures are repeated until a panel of conserved segments derived from all of the viral genomes in *G *is obtained. Assuming that nine 70-mer candidate conserved probes (*P *= {*p*_*j*_| *j *= 1,..., 9}) are selected by steps 5 and 6 in the probe design algorithm, Table 1 (Figure 2) demonstrates how to search for the minimal number of 70-mer genus probes that give similarity hits with all six viral members (*v*_*i*_| *i *= 1,..., 6) of *G*. Each "×" in Table 1 (Figure 2) indicates that *p*_*j *_has a similarity hit with *v*_*i*_. Based on the similarity-hit array (*A*_9 × 6_), at least two probes (*Card*(Ps*
 MathType@MTEF@5@5@+=feaafiart1ev1aaatCvAUfKttLearuWrP9MDH5MBPbIqV92AaeXatLxBI9gBaebbnrfifHhDYfgasaacH8akY=wiFfYdH8Gipec8Eeeu0xXdbba9frFj0=OqFfea0dXdd9vqai=hGuQ8kuc9pgc9s8qqaq=dirpe0xb9q8qiLsFr0=vr0=vr0dc8meaabaqaciaacaGaaeqabaqabeGadaaakeaacqWGqbaudaqhaaWcbaGaem4CamhabaGaeiOkaOcaaaaa@304D@)) are required for the full detection of all the viruses in *G*. Among the eight two-element probe sets (Ps*
 MathType@MTEF@5@5@+=feaafiart1ev1aaatCvAUfKttLearuWrP9MDH5MBPbIqV92AaeXatLxBI9gBaebbnrfifHhDYfgasaacH8akY=wiFfYdH8Gipec8Eeeu0xXdbba9frFj0=OqFfea0dXdd9vqai=hGuQ8kuc9pgc9s8qqaq=dirpe0xb9q8qiLsFr0=vr0=vr0dc8meaabaqaciaacaGaaeqabaqabeGadaaakeaacqWGqbaudaqhaaWcbaGaem4CamhabaGaeiOkaOcaaaaa@304D@ ≡ {(*p*_1_, *p*_3_), (*p*_1_, *p*_4_), (*p*_1_, *p*_5_), (*p*_1_, *p*_6_), (*p*_1_, *p*_7_), (*p*_1_, *p*_9_), (*p*_2_, *p*_4_), (*p*_3_, *p*_4_)}) that can collectively yield similarity hits with all the virus members of *G *((∪∀pj∈Ps*pj)=G′
 MathType@MTEF@5@5@+=feaafiart1ev1aaatCvAUfKttLearuWrP9MDH5MBPbIqV92AaeXatLxBI9gBaebbnrfifHhDYfgasaacH8akY=wiFfYdH8Gipec8Eeeu0xXdbba9frFj0=OqFfea0dXdd9vqai=hGuQ8kuc9pgc9s8qqaq=dirpe0xb9q8qiLsFr0=vr0=vr0dc8meaabaqaciaacaGaaeqabaqabeGadaaakeaadaqadaqaamaatafabaGaemiCaa3aaSbaaSqaaiabdQgaQbqabaaabaGaeyiaIiIaemiCaa3aaSbaaWqaaiabdQgaQbqabaWccqGHiiIZcqWGqbaudaqhaaadbaGaem4CamhabaGaeiOkaOcaaaWcbeqdcqWIQisvaaGccaGLOaGaayzkaaGaeyypa0Jafm4raCKbauaaaaa@3E02@), probe set (*p*_1_, *p*_4_) is the best selection since the two probes together maximize the similarity hits (max  = 10) with *G *and therefore increase the likelihood of detecting a virus. Subsequently, probe set (*p*_2_, *p*_5_, *p*_8_) is randomly selected for second-round coverage of *G *among the three probe sets (*p*_2_, *p*_5_, *p*_8_), (*p*_2_, *p*_7_, *p*_9_), and (*p*_2_, *p*_8_, *p*_9_) because all of them produce the same eight similarity hits with all the virus members of *G*.

We subsequently used the coronavirus genus to test the procedure for finding similar viral sequence segments. The SARS-CoV genome (*v*_SARS-CoV_) was aligned with the other 10 genomes *G*^(SARS-CoV) ^of the coronavirus genus using the BLASTN program (Fig. 1C). In the figure, each viral genome of the coronavirus genus is represented by a color-coded block, where the height of each pile of blocks along the SARS-CoV genome sequence represents the number of similarity hits (*S*_SARS-CoV_) with the other 10 genomes of the same genus for that particular sequence location (i.e., segment). The two viral segments showing the highest number of similarity hits with the other genomes (*C*_SARS-CoV_) were extracted for microarray probe design.

### *In silico *validation of the conserved sequence detection method

**Figure 3:**
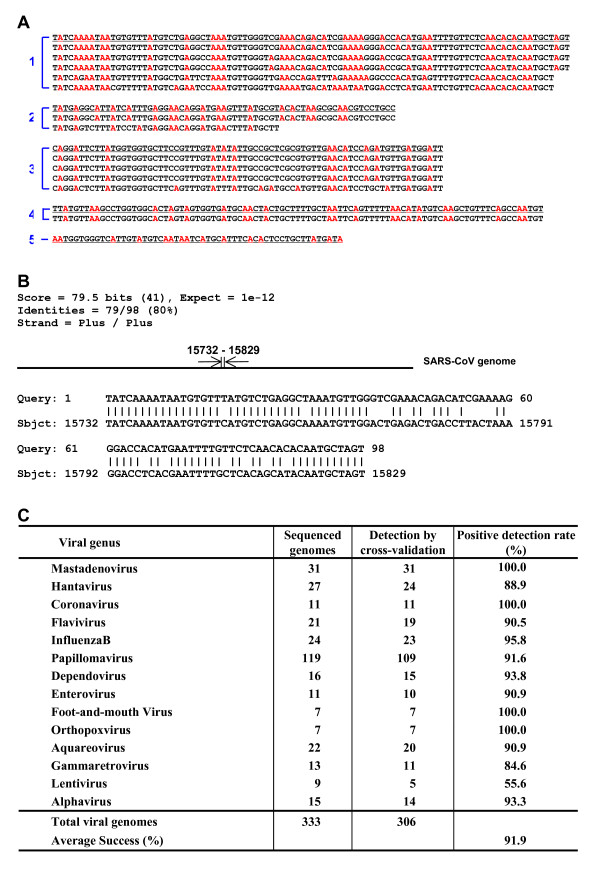
***In silico *validation of the conserved sequence design method**. All of the fully sequenced viral genomes (except that of SARS-CoV) in the coronavirus genus were used to determine the conserved sequences. The design method was validated by testing if the computed conserved sequences could successfully detect the novel SARS-CoV. (A) The redundant conserved segments as computed by steps 1 to 4 of the probe design algorithm were reduced to five nonredundant sequence groups by the BLASTN sequence alignment in step 5 of the probe design algorithm. The longest sequence (underlined sequence) in each group was used to represent the group. (B) BLASTN results for the comparison of the group 1 conserved sequence with the SARS-CoV genome. The BLASTN alignment outputs are shown in the figure. The sequence alignment with the SARS-CoV genome shows that the group 1 conserved sequence is located between nucleotides 15732 and 15829 of the SARS-CoV genome. (C) Larger-scale cross-validation of 14 viral genera consisting of 333 nonredundant sets of viral genomes.

To validate the method *in silico*, the SARS-CoV genome was removed from the coronavirus genus and treated as a test genome. The remaining 10 coronavirus viral genomes were used as the calibration set for computing the conserved sequences by the aforementioned procedures. To obtain high similarity genus probes, we used a high stringency criterion for hybrid formation in the conserved sequence detection method: 38-bp BLASTN sequence identity matches or >15 consecutive base pairing nucleotides [10]. Figure 3A shows the computed conserved sequence segment pool with redundancy (i.e., *C'*(*Coronavirus*^(SARS-CoV)^) in step 5 of the above probe design algorithm). These segments can be further sorted into five nonredundant groups by the BLASTN alignment program. The five longest sequences (underlined sequences in Fig. 3A) in each of the groups represent the nonredundant conserved sequence set of the coronavirus genus without SARS-CoV. A BLASTN sequence alignment between the SARS-CoV genome and the five nonredundant conserved sequences shows that the longest conserved sequence in group 1 had 80% (79/98) sequence similarity with the SARS-CoV genome at nucleotide positions from 15732 to 15829 (Fig. 3B) but no significant similarity with any genome in the other viral genera (data not shown).

To provide a more comprehensive *in silico *validation, we also performed leave-one-out cross-validation to the other viral members of the coronavirus genus and 13 other viral genera. In total, 14 viral genera and 333 nonredundant viral genome sequences were included in the large-scale cross-validation to verify the probe design algorithm (Fig. 3C). Based on a recent report that BLASTN sequence identity matches as short as 25-bp are sufficient to form a DNA hybrid [3], a lower hybrid formation criterion (25-bp vs. 38-bp BLASTN identity) was used to determine whether a test viral genome can be detected by a set of conserved sequences computed from the calibration genome set for a given genus. In Figure 3C, the column entitled "sequenced genomes" lists the number of viruses included for the computation in each genus, and the column entitled "detection by cross-validation" contains the number of viruses detected using the *in silico *method for each genus. The rightmost column is the positive detection rate for the *in silico *method for each genus. The computation results showed that most of the computed genus probes – especially for those genera having a larger number of sequenced viral genomes – displayed a good capability of detecting novel viruses, except for the genus of lentivirus, which yielded a worse prediction outcome. This result indicates that both the number of sequenced genomes and the extent of sequence conservation of viral members in a genus are useful factors for assessing the predictive power of the computed conserved sequences. Nevertheless, the *in silico *cross-validation results produced an average successful detection rate of 92%, which demonstrates the feasibility of the probe design method.

### Experimental verification

To experimentally verify the probe design method, we constructed a test virus chip with 72 70-mer oligonucleotides, which included genus probes covering 3 viral genera (7 conserved probes for coronavirus, 12 probes for flavivirus, and 7 probes for enterovirus) and specific probes covering 26 species of viruses in the coronavirus (7 species) and flavivirus (19 species) genera (see Additional File 2). A blank control (A549 lung adenocarcinoma cells) and seven viral samples [Japanese encephalitis virus (JEV), dengue virus types 2 (DEN-2), 3 (DEN-3), and 4 (DEN-4), SARS-CoV, enterovirus 71 (EV71), and a mixture of EV71 and SARS-CoV (EV71+SARS-CoV)], all of which are pathogens found in nasopharyngeal swabs, were used for the experiments.

**Figure 4:**
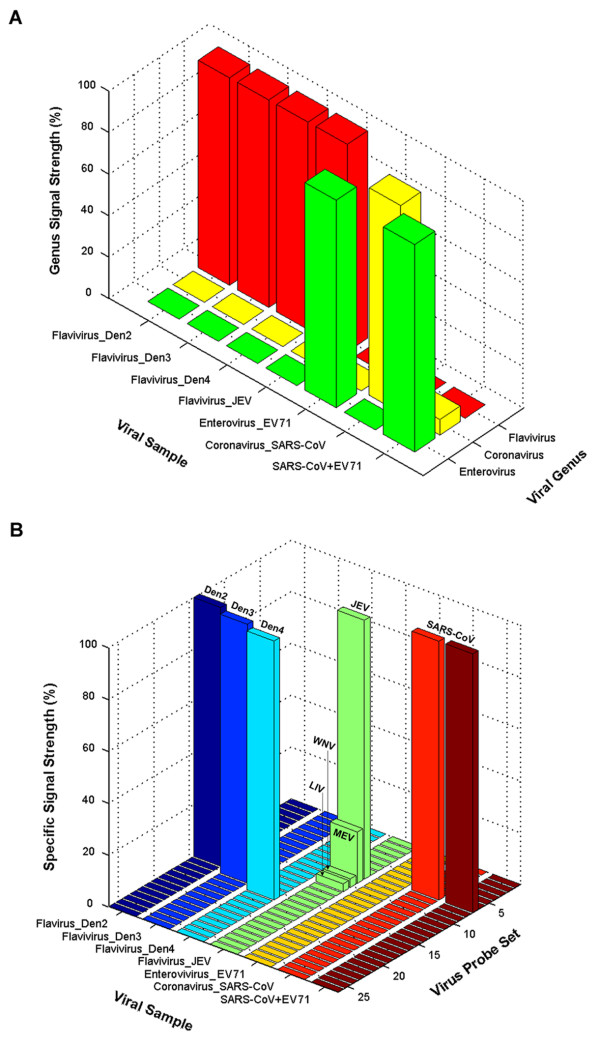
**Viral genus classification and specific virus identification**. Hybridization and identification results for seven test viral samples. The probes eligible for identity determination were prescreened using the hybridization intensity criteria described in the Methods section. (A) Viral genus determination for the seven viral samples, all of which belong to three viral genera. The genus signal strength is defined in the Methods section. (B) Determination of the identities of specific viruses. The numbers on the *Y*-axis are the ID numbers of the virus targets as listed in Additional File 2. Note that there are 46 species-specific probes for 26 viruses in the flavivirus and coronavirus genera, and that there are no enterovirus-specific probes.

The identity test results for the seven test samples are shown in Figure 4. The virus genus and species identification methods are described in the Methods section. In Figure 4A, the Z-axis represents the genus signal strengths of the three genus probe sets (*Y*-axis) hybridized to the seven test viral samples (*X*-axis). The possible genus identity (or identities) of a test viral sample can be determined from the magnitude of the genus signal. Figure 4B demonstrates the determination of the identity of a specific virus. The Z-axis is the specific signal strengths of 26 virus probe sets (*Y*-axis) hybridized to the 7 test viral samples (*X*-axis). The specific identity of the test virus can be read directly from the extent of the specific signal strength in the chart. Verifying the results for the genus-specific (Fig.4A) and cognate-species-specific (Fig. 4B) probes with virus taxonomy (i.e., the correct hierarchical genus and species association) results in the identities of all the test viruses being determined accurately and unambiguously. Figure 4 also demonstrates the use of this method for detecting emerging and unsequenced viruses. We intentionally left out EV71 sequence information in the conserved probe design process and made no EV71 species-specific probes on the test chip. However, the hybridization of EV71 to the chip resulted in a high signal intensity for the enterovirus genus probes, which clearly indicated that the virus belonged to this genus (Fig. 4A). Moreover, as shown by the hybridization result of the mixed viral sample EV71+SARS-CoV (Fig. 4A), the probe design method also allows two viruses of different genera to be accurately identified simultaneously. Although JEV cross-hybridized to three noncognate virus probes [Fig. 4B for Murray Valley encephalitis virus (MEV), West Nile virus (WNV), and Louping ill virus (LIV)] that are of the same genus as flavivirus, the signal strength resulting from the specific hybridization of JEV to its probe was 5-, 30-, and 33-fold higher than that of the probes for the three other viruses, respectively. Therefore, the virus was correctly identified as JEV. All the experiments were performed at least twice. In some cases, the signal intensity of the probes varied in different experiments for the same viral sample. However, our strategy for determining identities exhibits high signal-to-background ratios (as shown in Figure 4) and is both accurate and reproducible. Taken together, these results demonstrate the high specificity of the virus chip design.

## Discussion

The two conserved sequence fragments are highly similar to all of the coronavirus viral genomes archived in GenBank (Fig. 1C). This is not true in all cases: in general, most of the conserved sequences have significant similarity hits only with a fraction of the viral genomes in a genus. As demonstrated in Table 1 (Figure 2), the design method selects a panel of conserved genus probes, each one similar to several (but not all) of the viral genomes of a genus. The use of the whole panel of probes covers all the viral genomes of a genus. To enhance the capability of detecting emerging or unsequenced viruses, we created multiple probes sufficient for twofold coverage of the entire virus members in a genus.

The robustness of the computed genus probes is determined mainly by two parameters: (i) the number of existing fully sequenced viral genomes in a genus, and (ii) the degree of genome structure diversity for the viral members of the same genus. A larger number of available sequenced viral genomes in a genus makes it easier to identify the conserved sequences of the genus, and greatly enhances the probability of detecting unknown or unsequenced members of the genus. In addition, traditional virus taxonomy is not based on viral genome sequences. Thus, in some cases, the viral genomes are highly divergent among the members of a genus, such that each computed genus probe is only similar to a small fraction of the genus members, or may even be unique. The genus of lentivirus is one example, for which a poor detection rate (55.6%) based on *in-silico *cross-validation of the conserved genus sequences is shown in Figure 3C.

The principle behind the conserved probe design is to search for high similarity sequences that could hybridize to all the virus members of a given genus but not to the viruses of other genera. Therefore, accurate prediction of cross-hybridization is a prerequisite for the feasibility of the probe design method. In general, 70–80% global sequence similarity or a high local sequence similarity between two sequences could cause substantial cross-hybridization [10,14-16]. Significant cross-hybridization was reported for a nontarget 70-mer probe that had a (short) 25-bp BLASTN identity match [3]. To obtain as many reliable genus probes as possible, we used a 38-bp BLASTN sequence identity match criterion [10] in the genus probe design. This criterion was chosen to take into account both the global and the local sequence similarities in hybrid formation. Our experimental results show that the criterion reduces false-positive and -negative predictions of hybrid formation. Additionally, the number of genus probes sufficient for twofold coverage of all virus members in a genus ensures high detection accuracy in cases where the hybrid formation criterion may not apply. The experimental results shown in Figure 4 indicate that the hybrid formation criteria used in the genus probe design are sufficient to yield detectable genus probe signals to the target viruses but not to viruses of non-target genera.

The accurate detection of specific viruses by species-specific probes depends on avoiding nontarget cross-hybridization. Nevertheless, cross-hybridization to strains of viruses with extremely similar genome sequences or by confounding mechanisms may be inevitable. For example, to investigate the JEV cross-hybridization issue in Figure 4B, we performed BLASTN alignments for the cross-hybridizing virus probe sequences with the sequence of JEV, and found that none of the probe sequences had significant sequence similarity with JEV. Thus, the sequence complementarity guidelines do not explain why the JEV viral sample cross-hybridized to the other three non-target probes – this might instead be attributable to non-Watson-Crick base pairing [17-20].

In addition to the cross-hybridization issue, different probes for the same virus can yield very different signal intensities (e.g., the signal intensity for one probe was more than 100-fold lower than that for the other probe for the SARS-CoV virus, see Additional File 2). This may be attributed to the combination of the sequence-dependent effects of DNA hybridization [21,22] and the failure of random-primed PCR amplification to amplify the target regions of the probes.

Collectively, nucleic acid hybridization involves complex mechanisms that cannot yet be predicted purely by computation. Cross-hybridization and sequence-dependent variation in hybridization efficiency may be unavoidable in hybridization reactions involving a large number of probes, such as in the microarray method, making it desirable to have multiple specific probes for each virus. Viruses can be identified on the basis of the specific signal strength derived from the average signal intensity of multiple probes, since the cross-hybridization signals are always lower for the nontarget sequences than for the target sequences (which are a perfect match) [10,14-16,21].

There are, however, limitations to the virus microarray approach described here. An obvious one is that the range of viruses detectable by the method is limited by the content of viral genera archived in the database. Therefore, if the emerging new virus belongs to a heretofore unknown genus, then none of the probes would reveal a detectable hybridization signal.

Although our method ensures identification accuracy by verifying the results with the concordant taxonomical association of a genus and species, it is common for an assay sample to contain viruses of more than one genus or more than one species of viruses belonging to the same genus. As described in the Methods section, the viral genus is assigned using a scoring method based on signal strength; that is, *Sum*(*F*_*e*_|_*G*_)/max⁡G
 MathType@MTEF@5@5@+=feaafiart1ev1aaatCvAUfKttLearuWrP9MDH5MBPbIqV92AaeXatLxBI9gBaebbnrfifHhDYfgasaacH8akY=wiFfYdH8Gipec8Eeeu0xXdbba9frFj0=OqFfea0dXdd9vqai=hGuQ8kuc9pgc9s8qqaq=dirpe0xb9q8qiLsFr0=vr0=vr0dc8meaabaqaciaacaGaaeqabaqabeGadaaakeaadaWfqaqaaiGbc2gaTjabcggaHjabcIha4bWcbaGaem4raCeabeaaaaa@3222@(*Sum*(*F*_*e*_|_*G*_)). If more than one genus are present, there will be more than one non-zero *Sum(F*_*e*_|_*G*_*) *values. One of the genera will be given 100% and the others will be given a percentage smaller than 100%. Whether the signals are true genus signals or cross-hybridization signals can be verified by the presence of hybridization signals from the cognate species-specific probes. Only concordant genus and species identification results are accepted for assigning virus identities, which is one of the advantages of having separate probe sets for genus and species identification.

Moreover, the situation is more difficult than the above multigenus determination if more than one species belonging to the same genus are present,. Accurate discrimination of true signals from cross-hybridization depends on setting the correct signal thresholds together with discretion based on epidemiology statistics. High threshold values for the signal strengths increase the virus identification accuracy. As shown in Figure 4B, the cross-hybridization signals are several-fold lower than the true signal of JEV. Comparison with the results shown in Figure 4B reveals that the MEV, WNV, and LIV signals are false not only based on the threshold setting but also from the epidemiology statistics that coinfection or coexistence of any of these viruses in one assay sample is highly unlikely.

In this study we gathered virus information from GenBank which contains viral sequences of varying lengths, qualities and redundancy. It is noted that the viral sequences in GenBank are not well moderated [23] and taxonomic discrepancies exist among the curated viral databases available to the public, e.g., NCBI Viral Reference Genome collection [24] and the Universal Virus Database of International Committee on Taxonomy of Viruses (ICTVdB) [25]. This report describes an algorithm for identifying conserved genus sequences from a viral sequence database. The algorithm can be applied to any publicly available viral sequence database. However, for the purpose of computing conserved viral genus sequences, a large number of available sequences are needed and GenBank provides the most comprehensive collection of viral sequences available to the public. The large volume of data in GenBank fully satisfies the purpose of the present study: to develop a universal and comprehensive chip for identifying viruses, regardless of whether their genomes have been completely sequenced. The proposed approach of employing genus- and species-specific probe sets makes it possible to minimize the number of probes and cover all known viruses, including animal- and plant-infecting viruses, on a single chip.

## Conclusion

Most existing virus detection and identification methods are capable of identifying only a small number of known viruses in a single assay. Parallel screening for a broad spectrum of uncharacterized viruses using microarray technology overcomes this limitation and also provides a means of discovering emerging new viruses. Our probe design concept employs a panel of conserved sequence probes to directly classify emerging or unsequenced viruses at the genus level, and specific probes for known virus identification in a direct-readout manner. Moreover, using the two types of probes to determine the identity of a virus in a complementary way further assures detection accuracy. The ability to detect a large variety of viruses using the virus microarray approach has great potential in facilitating the discovery of emerging viruses and diagnosing diseases of unknown etiology or patients infected with unknown or multiple simultaneous viral pathogens.

In summary, we have established a virus probe design method and virus identification process for viral classification and identification based on a microarray approach. Our new probe design strategy and virus detection method allow direct reading of virus identity, and differ significantly from the probe design and virus detection methods described in the literature [2,26]. The concept and feasibility have been demonstrated using several example viruses. The methods and protocols can be applied to the analysis of large-scale virus microarray data to determine the identities of detected viruses by direct readout of a computer program.

Finally, the genera and species-specific probes for more than 5,700 viruses are archived in a database that is available on the Internet . To our knowledge, this database is the most comprehensive virus probe database currently available to the public. The use of the new probe design algorithm, which does not involve tiling probes, reduces the number of probes on a virus microarray yet retains high identification accuracy. The new probe design method makes it feasible to construct a virus microarray with sufficient probes for identifying all the sequenced viruses that infect animals and plants.

## Methods

### Virus identity determination

We developed the following identification procedure to compute the identity of a virus directly from the hybridization signals. First, to minimize nonspecific hybridization, any probe with a fluorescence signal intensity, *F*, satisfying the following two criteria is eligible to use in the subsequent viral identification procedure: (i) *F*_*viral sample*_/*F*_*blank *_≥ 3 and (ii) *F*_*viral sample *_– *F*_*blank *_> 1500. To take both the number and signal intensity of probes satisfying the above criteria in a given genus probe set into consideration, the following genus signal strength (defined as a percentage) is used to determine the viral genus identity: *Sum*(*F*_*e*_|_*G*_)/max⁡G
 MathType@MTEF@5@5@+=feaafiart1ev1aaatCvAUfKttLearuWrP9MDH5MBPbIqV92AaeXatLxBI9gBaebbnrfifHhDYfgasaacH8akY=wiFfYdH8Gipec8Eeeu0xXdbba9frFj0=OqFfea0dXdd9vqai=hGuQ8kuc9pgc9s8qqaq=dirpe0xb9q8qiLsFr0=vr0=vr0dc8meaabaqaciaacaGaaeqabaqabeGadaaakeaadaWfqaqaaiGbc2gaTjabcggaHjabcIha4bWcbaGaem4raCeabeaaaaa@3222@(*Sum*(*F*_*e*_|_*G*_)), where *F*_*e*_|_*G *_represents the hybridization intensity of any eligible genus probe in a genus *G*, *Sum*(*F*_*e*_|_*G*_) is the sum of all the *F*_*e*_|_*G *_in *G*, and max⁡G
 MathType@MTEF@5@5@+=feaafiart1ev1aaatCvAUfKttLearuWrP9MDH5MBPbIqV92AaeXatLxBI9gBaebbnrfifHhDYfgasaacH8akY=wiFfYdH8Gipec8Eeeu0xXdbba9frFj0=OqFfea0dXdd9vqai=hGuQ8kuc9pgc9s8qqaq=dirpe0xb9q8qiLsFr0=vr0=vr0dc8meaabaqaciaacaGaaeqabaqabeGadaaakeaadaWfqaqaaiGbc2gaTjabcggaHjabcIha4bWcbaGaem4raCeabeaaaaa@3222@(*Sum*(*F*_*e*_|_*G*_)) represents the maximum value of *Sum*(*F*_*e*_|_*G*_) among all the viral genera represented on a microarray. The viral genus identity in a test sample is assigned to the genus with the highest genus signal strength (i.e., 100%). The presence of multiple viruses of different genera in a sample will result in a nonzero genus signal strength. We then score and rank the possible virus identities in the specific probe region on the basis of the *specific signal strength*, defined as the average signal intensity of the multiple probes for a given virus divided by the maximum average signal intensity of the multiple probes for every virus represented on a microarray (expressed as a percentage). As for the genus determination method, the presence of multiple viruses in the sample will result in a nonzero signal strength. Finally, the identity of the virus is established by the concordant results for both the genus- and cognate-species-specific probes.

### Array fabrication

Oligonucleotides of 70 mers were synthesized and modified with 5' amino-linker moieties by standard phosphoramidite-synthesis chemistry on a homemade DNA synthesizer [27], and desalted by Sephadex (G50 DNA grade; Amersham, NJ). The 70-mer oligonucleotide probes were dissolved in Pronto! Epoxide spotting solution (Corning, NY) at a concentration of 40 μM and spotted on Corning Epoxide glass slides using a homemade arrayer. The slides were processed according to the manufacturer's instructions.

### Cell culture and viruses

The JEV (RP-9 strain, GenBank accession number: AF014161) and DEN-2 (PL046 strain), DEN-3 (H87 strain), and DEN-4 (H241 strain) used in this study were propagated in C6/36 cells using RPMI 1640 medium containing 5% fetal bovine serum (FBS) (Invitrogen, CA). The EV71 (*neu *strain) stock was propagated in RD cells cultured in DMEM (Dulbecco's modified Eagle's medium) supplemented with 2% FBS, penicillin (100IU/ml), and streptomycin (100 IU/ml). SARS-CoV RNA was kindly provided by the Taiwan Center for Disease Control. The copy number of SARS-CoV genomes in a sample was measured using a commercially available quantitative RT-PCR kit (RealArt™ HPA-Coronavirus LC RT PCR Reagents, ARTUS, Germany). The amount of viruses used in the study were as follows: (i) 1.75 × 10^4 ^pfu of DEN-2, (ii) 3.5 × 10^3 ^pfu of DEN-3, (iii) 1.75 × 10^3 ^pfu of DEN-4, (iv) 13 μg of total RNA derived from JEV-infected A549 lung adenocarcinoma cells, (v) 1.5 × 10^4 ^copy numbers of SARS-CoV, (vi) 2.33 × 10^3 ^pfu of EV7, and (vii) a mixture of 2.33 × 10^3 ^pfu of EV71 and 100 copy numbers of the SARS-CoV viral genome. The blank control was 11.2 μg of total RNA derived from A549 lung adenocarcinoma cells.

### Sample amplification, labeling, hybridization, and data analysis

The viral genomic sequences were randomly amplified as described previously [2,28]. The amplified PCR product was purified with the MiniElute PCR purification kit (Qiagen, CA). Cy3-dUTP was incorporated into the purified PCR product with random octamers using the Klenow fragment of DNA polymerase I. The hybridization of the Cy3-labeled DNA fragments to the Corning Epoxide slide was performed at 42°C for 16–18 hr in Pronto! microarray hybridization buffer (Corning) under an 18 × 18 mm coverslip in a sealed chamber. After hybridization, the arrays were washed three times with Pronto! washing reagents. After drying by centrifugation, the arrays were scanned with a GenePix 4000B scanner (Axon Instruments, CA). Array image acquisition and signal analysis were performed using GenePix Pro 4.0 software.

## Authors' contributions

This study was conceived by CCC, KP, and PCY. CCC devised the algorithm, performed the computer programming, and designed the experimental strategy, with guidance from KP and PCY. TTL also worked on the programming and constructed the virus database. CHC was responsible for the mathematical formulation and data analysis. YLL and MSH prepared virus cultures as well as viral genomic materials and drafted the parts of the text related to virology. HYH designed and performed the microarray experiments and data analysis. CCC drafted the manuscript and revisions were made by KP and PCY. All authors read and approved the final manuscript.

## Supplementary Material

Additional File 1 **Example description of step 5 of the probe design algorithm**. This file contains a description of step 5 of the probe design algorithm. Example viruses are used to illustrate how this step identifies conserved sequences for a viral genus. Click here for file

Additional File 2 **Virus probe sequences on the test chip, and hybridization data of the probes to various samples**. This file contains information on the probes and experimental hybridization data of the test chips used in an empirical approach to demonstrate the validity of the probe design algorithm. Click here for file

## References

[B1] Elnifro EM, Ashshi AM, Cooper RJ, Klapper PE (2000). Multiplex PCR: optimization and application in diagnostic virology. Clin Microbiol Rev.

[B2] Wang D, Coscoy L, Zylberberg M, Avila PC, Boushey HA, Ganem D, DeRisi JL (2002). Microarray-based detection and genotyping of viral pathogens. Proc Natl Acad Sci USA.

[B3] Wang D, Urisman A, Liu YT, Springer M, Ksiazek TG, Erdman DD, Mardis ER, Hickenbotham M, Magrini V, Eldred J, Latreille JP, Wilson RK, Ganem D, DeRisi JL (2003). Viral discovery and sequence recovery using DNA microarrays. PLoS Biol.

[B4] Sengupta S, Onodera K, Lai A, Melcher U (2003). Molecular detection and identification of influenza viruses by oligonucleotide microarray hybridization. J Clin Microbiol.

[B5] Kessler N, Ferraris O, Palmer K, Marsh W, Steel A (2004). Use of the DNA flow-thru chip, a three-dimensional biochip, for typing and subtyping of influenza viruses. J Clin Microbiol.

[B6] Chua KB, Bellini WJ, Rota PA, Harcourt BH, Tamin A, Lam SK, Ksiazek TG, Rollin PE, Zaki SR, Shieh W, Goldsmith CS, Gubler DJ, Roehrig JT, Eaton B, Gould AR, Olson J, Field H, Daniels P, Ling AE, Peters CJ, Anderson LJ, Mahy BW (2000). Nipah virus: a recently emergent deadly paramyxovirus. Science.

[B7] Rota PA, Oberste MS, Monroe SS, Nix WA, Campagnoli R, Icenogle JP, Penaranda S, Bankamp B, Maher K, Chen MH, Tong S, Tamin A, Lowe L, Frace M, DeRisi JL, Chen Q, Wang D, Erdman DD, Peret TC, Burns C, Ksiazek TG, Rollin PE, Sanchez A, Liffick S, Holloway B, Limor J, McCaustland K, Olsen-Rasmussen M, Fouchier R, Gunther S, Osterhaus AD, Drosten C, Pallansch MA, Anderson LJ, Bellini WJ (2003). Characterization of a novel coronavirus associated with severe acute respiratory syndrome. Science.

[B8] Urisman A, Fischer KF, Chiu CY, Kistler AL, Beck S, Wang D, DeRisi JL (2005). E-Predict: a computational strategy for species identification based on observed DNA microarray hybridization patterns. Genome Biol.

[B9] Chang PC, Peck K (2003). Design and assessment of a fast algorithm for identifying specific probes for human and mouse genes. Bioinformatics.

[B10] Kane MD, Jatkoe TA, Stumpf CR, Lu J, Thomas JD, Madore SJ (2000). Assessment of the sensitivity and specificity of oligonucleotide (50 mer) microarrays. Nucleic Acids Res.

[B11] Zuker M, Mathews DH, Tuner DH, Barciszewski J, Clark BFC (1999). Algorithms and Thermodynamics for RNA Secondary Structure Prediction: A Practical Guide. RNA Biochemistry and Biotechnology.

[B12] Cormen TH, Leiserson CE, Rivest RL, Stein C (2001). Introduction to algorithms.

[B13] Johnson DS (1974). Approximation algorithms for combinatorial problems. J Comput System Sci.

[B14] Evertsz EM, Au-Young J, Ruvolo MV, Lim AC, Reynolds MA (2001). Hybridization cross-reactivity within homologous gene families on glass cDNA microarrays. Biotechniques.

[B15] Hughes TR, Mao M, Jones AR, Burchard J, Marton MJ, Shannon KW, Lefkowitz SM, Ziman M, Schelter JM, Meyer MR, Kobayashi S, Davis C, Dai H, He YD, Stephaniants SB, Cavet G, Walker WL, West A, Coffey E, Shoemaker DD, Stoughton R, Blanchard AP, Friend SH, Linsley PS (2001). Expression profiling using microarrays fabricated by an ink-jet oligonucleotide synthesizer. Nat Biotechnol.

[B16] Xu W, Bak S, Decker A, Paquette SM, Feyereisen R, Galbraith DW (2001). Microarray-based analysis of gene expression in very large gene families: the cytochrome P450 gene superfamily of Arabidopsis thaliana. Gene.

[B17] Chou SH, Chin KH (2001). Solution structure of a DNA double helix incorporating four consecutive non-Watson-Crick base-pairs. J Mol Biol.

[B18] Kaderali L, Schliep A (2002). Selecting signature oligonucleotides to identify organisms using DNA arrays. Bioinformatics.

[B19] Karthikeyan G, Wagle MD, Rao BJ (1998). Non-Watson-Crick base pairs modulate homologous alignments in RecA pairing reactions. FEBS Lett.

[B20] Wong K-Y, Vainrub A, Powdrill T, Hogan M, Pettitt BM (2004). A non-Watson-Crick motif of base-pairing on surfaces for untethered oligonucleotides. Mol Simulation.

[B21] Chou CC, Chen CH, Lee TT, Peck K (2004). Optimization of probe length and the number of probes per gene for optimal microarray analysis of gene expression. Nucleic Acids Res.

[B22] Holloway AJ, van Laar RK, Tothill RW, Bowtell DD (2002). Options available–from start to finish–for obtaining data from DNA microarrays II. Nat Genet.

[B23] Check E (2006). Powerful new database pins down emerging infections. Nat Med.

[B24] Bao Y, Federhen S, Leipe D, Pham V, Resenchuk S, Rozanov M, Tatusov R, Tatusova T (2004). National center for biotechnology information viral genomes project. J Virol.

[B25] Buchen-Osmond C (1997). Further progress in ICTVdB, a universal virus database. Arch Virol.

[B26] Allander T, Emerson SU, Engle RE, Purcell RH, Bukh J (2001). A virus discovery method incorporating DNase treatment and its application to the identification of two bovine parvovirus species. Proc Natl Acad Sci USA.

[B27] Cheng JY, Chen HH, Kao YS, Kao WC, Peck K (2002). High throughput parallel synthesis of oligonucleotides with 1536 channel synthesizer. Nucleic Acids Res.

[B28] Bohlander SK, Espinosa R, Le Beau MM, Rowley JD, Diaz MO (1992). A method for the rapid sequence-independent amplification of microdissected chromosomal material. Genomics.

