# Supplementary material for: Design of microarray probes for virus identification and detection of emerging viruses at the genus level
Source: BMC Bioinformatics. 2006 Apr 28;7:232. doi: 10.1186/1471-2105-7-232 (PMC1523220; doi:10.1186/1471-2105-7-232)
Supplement: Additional File 1 — Example description of step 5 of the probe design algorithm. This file contains a description of step 5 of the probe design algorithm. Example viruses are used to illustrate how this step identifies conserved sequences for a viral genus. [file 1471-2105-7-232-S1.doc]

# Example description of step 5 of the probe design algorithm

Assuming that there are four viruses {*v_i_| i*=1,…, 4} in a viral genus *G*, the similarity sequence segments of the four viruses can be depicted as follows:

.

**Figure 1**

The horizontal axes in Figure 1 represent the query sequences. The viral sequences are aligned with the query sequence by the BLASTN program, and only the segments sharing significant sequence similarity with the query sequence are shown in the vertical axis. Figure 1A illustrates that virus *v*_1_ contains a segment that shares significant sequence similarity with viruses *v*_2_ and *v*_3_, and this segment is designated as segment 1 with the position marked in red. The same procedure applies to viruses *v*_2_, *v*_3_, and *v*_4_. Figure 1B and 1C show that segment 1 is the common segment in both alignments. However, in Figure 1C, once *v*_4_ is included in the conserved sequence computation, a shorter conserved segment (segment 1’, which is a partial fragment of segment 1) is generated since the similarity sequence segment between *v*_3_ and *v*_4_ only partially overlaps with segment 1. Because the genome organization of *v*_4_ differs from that of the others, the computed conserved sequence for *v*_4_ (red segment 2 in Figure 1D) is also different from conserved segment 1.

Collectively, the conserved sequence set of the viral genus, *C'*(*G*) as defined in the step 5, contains segments 1, 1’, and 2. It is apparent that sequence redundancy exists in *C'*(*G*) since segment 1’ is a partial fragment of segment 1. To eliminate this redundancy, the longest conserved sequence (*C_L_*, segment 1 in the example) in *C'*(*G*) is selected first and aligned against the others (*C'*(*G*)–{*C_L_*}, segments 1’ and 2) by BLASTN. A sequence segment would be grouped with the longest one (segment 1) if it has ≥80% sequence similarity (with respect to the length of the sequence segment) with the longest one. In this example, segments 1 and 1’ would be grouped together to form the first subgroup (*C'*_1_(*G*)), and the longest stretch (segment 1) is renamed as *C*_(1)_ to represent the first subgroup. The above procedure is repeated for the remaining sequence segments (segment 2) in *C'*(*G*) until every sequence in *C'*(*G*) is assigned to one subgroup. In this example, segments 1 and 1’ are grouped together (*C'*_1_(*G*)) and segment 2 (*C*_(2)_) resides in another subgroup by itself (*C'*_2_(*G*)). Thus, segments 1 and 2 constitute the nonredundant conserved sequence set of viral genus *G* ($\cup_{i=1}^{k} C_{(i)}$). The above example is a simplified version of the second conserved sequence group in Figure 2A of the manuscript.

It is noted that several subdatabases are used in the algorithm. In the above example, the *G* database contains the viral genomes (*v*_1_, *v*_2_, *v*_3_, *v*_4_) of a genus downloaded from GenBank. Virtual subdatabase *G*(*^i^*) is the viral genome database for one viral genus without the query genome. In the example, if *v*_1_ is the query genome, then (*v*_2_, *v*_3_, *v*_4_) forms subdatabase *G*(*^i^*). The above figure, combined with steps 1 to 4 of the algorithm as depicted by Figure 1A and 1B in the manuscript, as well as the above descriptions, collectively show how the *C'*(*G*) subdatabase is derived from the *G* database and how to obtain the nonredundant conserved sequence subdatabase of *C'*(*G*), $\cup_{i=1}^{k} C_{(i)}$.
